# Underwater endoscopic submucosal dissection with nasal intubation using a small-bore tracheal tube for an early epiglottic lesion

**DOI:** 10.1055/a-2445-8287

**Published:** 2024-12-03

**Authors:** Hui Wang, Jingjing Yao, Yang Liu, Feifei Zhang, Yuehong Qiu, Wen Jiao, Jindong Fu

**Affiliations:** 1 549615 Gastroenterology, Rizhao People’s Hospital, Rizhao, China

A 60-year-old man was diagnosed during gastroscopy with an early neoplastic lesion on the lingual surface of the epiglottis. He had a history of long-term alcohol consumption. The lesion, measuring approximately 1.5 × 2.0 cm, was identified as a superficial, flat (0-IIb) area with a clear boundary, exhibiting a reddish hue under white light ( Fig. 1 **a** ) and appearing brown under narrow-band imaging (NBI) ( Fig. 1 **b** ). Following biopsy, histopathological examination confirmed high grade intraepithelial neoplasia (HGIN), and computed tomography (CT) scans showed no evidence of metastasis.

**Fig. 1:**
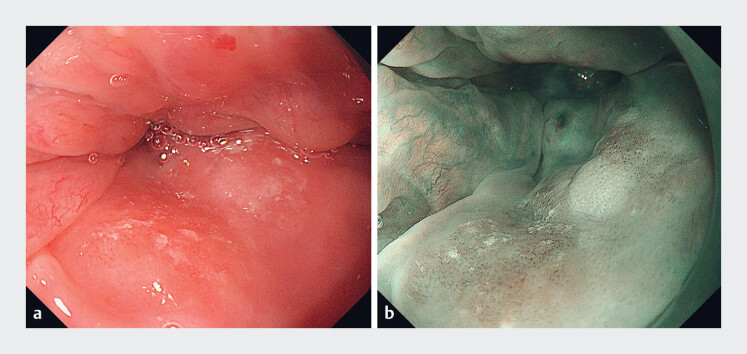
Endoscopic images of an early neoplastic lesion on the lingual surface of the epiglottis: **a** superficial flat lesion, exhibiting a reddish hue under white light; **b** brown-colored appearance under narrow-band imaging.

After informed consent had been obtained, the patient underwent endoscopic submucosal dissection (ESD) with tracheal intubation, facilitated by the nasal insertion of a 6.0-mm small-bore tracheal tube ( Video 1 ). The lesion was distinctly marked under magnifying endoscopy. Given the confined space of the epiglottis, identifying the optimal dissection layer was challenging. To address this, following a circumferential mucosal incision performed using a Goldknife (Micro-tech, Nanjing, China) ( Fig. 2 **a** ), the dissection was performed using the water immersion method, which effectively exposed the dissection plane ( Fig. 2 **b, c** ). The procedure was executed successfully without complications such as bleeding or perforation ( Fig. 2 **d** ). Postoperative pathology confirmed HGIN with negative resection margins. At 1 month post surgery, the patient showed a satisfactory recovery with normal swallowing function preserved.

**Fig. 2:**
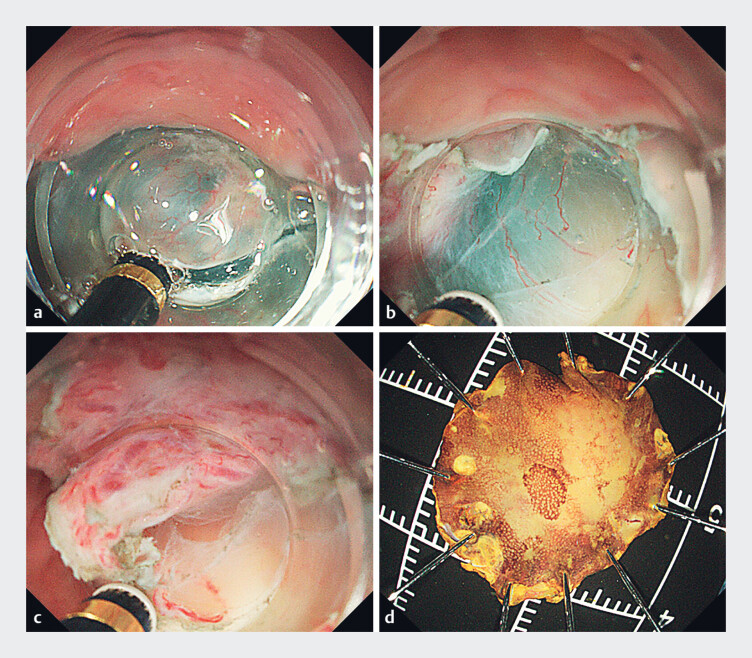
Underwater endoscopic submucosal dissection (ESD) procedure. **a** Circumferential mucosal incision of the lesion. **b,c** Dissection performed using the water immersion method. **d** Macroscopic appearance of the resected lesion.

ESD has emerged in recent years as an effective and less invasive treatment for early pharyngeal lesions, enhancing patients’ postoperative quality of life 1 . The limited space of the epiglottic region presents unique challenges for ESD. In this case, the use of a small-bore tracheal tube for nasal intubation minimized the space occupied, and the underwater method was applied to leverage the buoyancy and magnification effects of water, enhancing the visibility of the dissection plane and improving the efficiency and safety of the procedure 2 . This case represents the first reported instance of underwater ESD for an early neoplastic lesion in the epiglottic region, demonstrating the safety and efficacy of the technique for such lesions.

Underwater endoscopic submucosal dissection (ESD), with nasal intubation using a small-bore tracheal tube, performed for an early epiglottic lesion.Video 1

Endoscopy_UCTN_Code_TTT_1AO_2AG_3AD

## Notes

Endoscopy E-Videos https://eref.thieme.de/e-videos
*E-Videos* is an open access online section of the journal *Endoscopy* , reporting on interesting cases and new techniques in gastroenterological endoscopy. All papers include a high-quality video and are published with a Creative Commons CC-BY license. Endoscopy E-Videos qualify for HINARI discounts and waivers and eligibility is automatically checked during the submission process. We grant 100% waivers to articles whose corresponding authors are based in Group A countries and 50% waivers to those who are based in Group B countries as classified by Research4Life (see: https://www.research4life.org/access/eligibility/ ). This section has its own submission website at https://mc.manuscriptcentral.com/e-videos .
